# Disease control efficacy of 32,33-didehydroroflamycoin produced by *Streptomyces rectiviolaceus* strain DY46 against gray mold of tomato fruit

**DOI:** 10.1038/s41598-019-49779-6

**Published:** 2019-09-19

**Authors:** Jeong Do Kim, Min Young Park, Byeong Jun Jeon, Beom Seok Kim

**Affiliations:** 1 Korea Institute of Science and Technology (KIST) Gangneung Institute, Gangneung, 25451 Republic of Korea; 2 Department of Biosystems and Biotechnology, Korea University Graduate School, Seoul, 02841 Republic of Korea; 3 Division of Biotechnology, College of Life Sciences and Biotechnology, Korea University, Seoul, 02841 Republic of Korea

**Keywords:** Antifungal agents, Applied microbiology

## Abstract

Despite the efficacy of synthetic fungicides in controlling postharvest diseases, public concerns regarding chemical residues in food and an increase in drug-resistant strains of pathogens have led to a need for new agents to control postharvest diseases. The current study was performed to find control agents of microbial origin that are effective on gray mold of tomato fruits. We recently isolated *Streptomyces rectiviolaceus* DY46, which has antagonistic activity against various plant pathogenic fungi. The incidence of gray mold of tomato fruits was markedly reduced by 80.0% in tomatoes treated with the cell extract of *Streptomyces rectiviolaceus* DY46 compared with the control tomatoes. The active ingredient was purified from the cell extract of DY46 and identified to be 32,33-didehydroroflamycoin (DDHR). DDHR displayed MICs (minimal inhibitory concentrations) against the mycelial growth of various plant pathogenic fungi at concentrations of 8–64 mg L^−1^. The incidence of gray mold in tomato fruits inoculated with conidial suspension (10^4^ conidia mL^−1^) of *Botrytis cinerea* was markedly reduced by 88.9% in tomatoes treated with DDHR (100 mg L^−1^) compared with the control. The DDHR residue in tomato fruit was significantly diminished 2 d after treatment. These results show that DDHR would be relatively safe for use as a postharvest fungicide.

## Introduction

Tomato (*Solanum lycopersicum* L.), which is rich in powerful antioxidants, such as carotenoids, vitamins C and E, and flavonoids, is one of the most economically important vegetable crops cultivated worldwide^1–3^. Gray mold caused by *Botrytis cinerea* is a devastating disease of tomato fruit during the pre- and postharvest periods, which results in quality deterioration and a severe decrease in marketable yield^4^. *B. cinerea* infects all aerial parts of tomato plants and green and mature fruits^5^. In infected tomato fruit, water-soaked lesions rapidly expand and spread to adjacent healthy fruit by direct contact with the infected fruit during storage^6^. Postharvest management of gray mold is particularly challenging. Cold storage alone cannot sufficiently control gray mold because *B. cinerea* can grow, sporulate, and proliferate even at temperatures as low as −0.5 °C^4,7^. Synthetic fungicides are a common and effective means to prevent the deterioration in quality and decline in marketable yield caused by postharvest diseases and to extend the shelf-life of fruit and vegetables^6,8^. However, only a few fungicides have been approved for use as control agents for postharvest diseases due to strict regulatory policies for food safety^4,6,8–10^. In addition to public concerns regarding food safety, there is an occurrence of fungicide-resistant strains; therefore, new agents for controlling postharvest diseases are required^11,12^.

Microbial-derived natural products are a promising source for developing new postharvest fungicides. Microorganisms can synthesize versatile metabolite structures that can interact with diverse biological targets, and the metabolites are usually degraded within a month or even a couple of days when exposed to the environment, thereby leading to low residual levels that are less harmful in an exposed ecosystem^13,14^.

We recently isolated *Streptomyces* sp. strain DY46 from soil samples collected from Danyang Province, Korea. The strain exhibited the ability to suppress gray mold of tomato fruit. In the present study, the polyene macrolide 32,33-didehydroroflamycoin (DDHR) was purified and identified from the cell extract of DY46 using various chromatographic and spectroscopic methods. In addition, we evaluated the antifungal activity of DDHR against various plant pathogenic fungi and its ability to control gray mold of tomato fruits.

## Results

### Isolation and identification of strain DY46

The strain DY46 formed aerial mycelia and spores typical of strains belonging to the genus *Streptomyces*. As observed by scanning electron microscopy (SEM), the spore chains of strain DY46 were rectiflexible and consisted of cylindrical, smooth-surfaced spores with a diameter of 0.6–0.9 µm (Fig. S1). A partial 16S rDNA (1361 bp) sequence of DY46 (GenBank Accession Number: KP771698) showed 99% identity with that of *Streptomyces rectiviolaceus* strain NBRC 100765 (GenBank Accession Number: NR112590). Specific structures, such as synnemata, sclerotia, or sporangia, were not observed. Based on these results, strain DY46 was identified as *S. rectiviolaceus*.

### Evaluation of disease control efficacy of the cell extract of strain DY46

**Figure 1:**
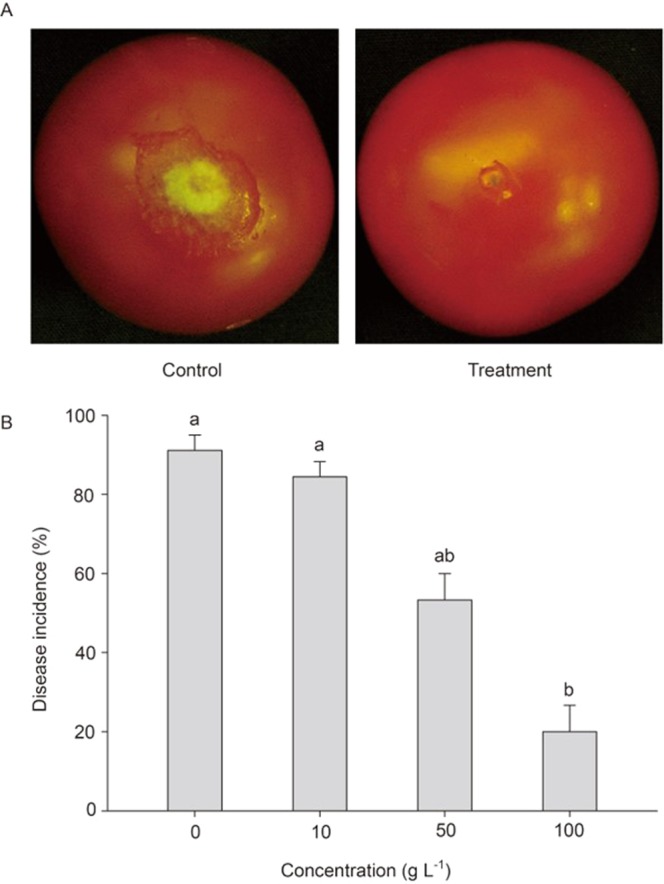
The disease control efficacy of the cell extract of the DY46 strain against gray mold of tomato fruit. Tomato fruits were treated with water containing 1% methanol and cell extract of DY46 (**A**). The disease incidence (%) in tomato fruits treated with the cell extract of DY46 (**B**). Disease incidence (%) was evaluated 2 d after fungal inoculation. Bars represent the standard deviation of three replicate experiments. Lowercase letters beside the bars indicate significant differences between treatments according to the least significant difference test (n = 15; *P* < 0.05).

The disease control efficacy of the cell extract of DY46 against gray mold was evaluated on tomato fruits 2 d after inoculation with *B. cinerea*. The disease incidence of control fruits treated with 5 μL of water containing 1% methanol was 91.1%, while the disease incidence of the tomato fruits treated with the cell extract diminished in a dose-dependent manner. Notably, the disease incidence was significantly reduced by 80% in the tomato fruits treated with 100 g L^−1^ of the cell extract compared with the control (Fig. 1). The cell extract did not have any apparent detrimental effect on the tomato fruits.

### Active ingredient in the cell extract of strain DY46

A series of chromatographic procedures were performed to purify the active ingredient in the crude cell extract of DY46. The fractions of 100% methanol and acetone eluates obtained from Diaion HP-20 column chromatography showed antifungal activity against *B. cinerea*. The active fractions were pooled and subjected to C18 column chromatography for further purification. The fractions of 80% methanol eluates showing antifungal activity were pooled and concentrated *in vacuo* and then subjected to a semipreparative HPLC equipped with a C18 reverse-phase column. The active ingredient eluted at a retention time of 62.9 min was denoted as DY46A. The yield of DY46A was 2.6 mg of a yellow amorphous substance after being dried *in vacuo*.

### Structure of DY46A

**Figure 2:**
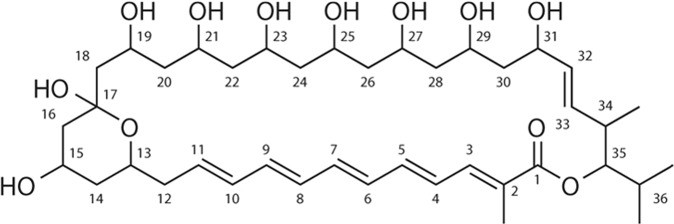
The structure of DY46A (32,33-didehydroroflamycoin) from *Streptomyces rectiviolaceus* strain DY46.

DY46A had a characteristic UV absorption pattern with maximum absorbance at 263 and 365 nm and a shoulder at 375 nm, which implied a conjugated pentaene structure. The molecular formula of DY46A was determined using HRMS data [DY46A (M^−^H)^−^; calcd: 735.4320; found: 735.4336] as C_40_H_64_O_12_ (Fig. S2). The ^1^H NMR spectrum showed a number of olefinic, methylene, and oxymethine protons along with one singlet methyl signal at 1.97 (3H, s) and three doublet methyl signals at δ_H_ 0.88 (3H, d), 0.99 (3H, d), and 1.08 (3H, d). ^13^C NMR and HMBC spectra of DY46A showed 40 signals, of which 10 were assigned to methylene, 23 to methine, 4 to methyl groups and 3 to quaternary carbons, including an oxygenated quaternary carbon and a carbonyl carbon belonging to lactone, on the basis of their chemical shifts. The nine *sp*^2^ methine carbon signals at δ_C_ 128.8, 132.2, 133.6, 133.7, 135.5, 137.3, 138.5, 140.1, and 141.8 were due to the pentaene structure. The other two *sp*^2^ methine carbon signals appearing at δ_C_ 133.3 corresponded to one double bond **(**Table S1**)**. Based on HRMS and NMR data, the structure of DY46A was found to correspond to that of 32,33-didehydroroflamycoin (DDHR) (Fig. 2). The spectroscopic data of DY46A were in perfect accord with those described previously for DDHR by Stodůlková *et al*.^15^.

### MIC of DDHR

*In vitro* antifungal spectra of DDHR were evaluated using a modified CLSI broth microdilution method (M38-A Document)^14,16^. The mycelial growth of all plant pathogenic fungi examined was inhibited by DDHR, except the oomycete pathogen *Phytophthora capsici*. DDHR inhibited the growth of *A. brassicicola*, *A. mali*, *B. cinerea*, *C. cucumerinum*, *C. coccodes*, *C. gloeosporioides*, *C. orbiculare*, *C. destructans*, *F. oxysporum* f. sp. *cucumerinum*, *F. oxysporum* f. sp. *lycopersici*, *P. expansum*, and *R. stolonifer* var. *stolonifer* at concentrations of 8–64 mg L^−1^. A polyene macrolide nystatin used as a positive control inhibited the mycelial growth of these fungi within the concentration range of 1–64 mg L^−1^ (Table 1).

**Table 1:** Minimum inhibitory concentrations (MICs) of DY46A against various plant pathogenic fungi.

Plant pathogenic fungi	MIC^a^ (mg L^−1^)	
	DY46A (DDHR)^c^	Nystatin
*Alternaria brassicicola*	8	1
*Alternaria mali*	16	1
*Botrytis cinerea*	32	1
*Cladosporium cucumerinum*	64	1
*Colletotrichum coccodes*	8	8
*Colletotrichum gloeosporioides*	8	8
*Colletotrichum orbiculare*	16	16
*Cylindrocarpon destructans*	32	64
*Fusarium oxysporum* f. sp. *cucumerinum*	16	4
*Fusarium oxysporum* f. sp. *lycopersici*	16	16
*Penicillium expansum*	16	1
*Phytophthora capsici*	>128^*b*^	>128
*Rhizopus stolonifer* var. *stolonifer*	8	4

^a^The lowest concentration that completely inhibited the growth of the plant pathogen was determined after incubation for 1–2 d.

^b^The growth of the tested microorganism was not inhibited at concentrations up to 128 mg L^−1^.

^c^DY46A was identified as 32,33-didehydroroflamycoin (DDHR).

### Efficacy of controlling gray mold development

The efficacy of DDHR in controlling gray mold was evaluated on tomato fruit and compared with that of the commercial fungicide fludioxonil (Table 2). Fludioxonil [Environmental Protection Agency (EPA) Reg. No. 100–759] was registered by the U.S. EPA as an agent for controlling postharvest disease of tomato in 2012. Two days after infection, control fruit showed 100% disease incidence. However, treatments with 10 mg L^−1^ of fludioxonil and 100 mg L^−1^ of DDHR significantly reduced disease incidence to 22.2% and 14.8%, respectively. Fludioxonil completely protected tomato fruits from gray mold infection at 100 mg L^−1^. The incidence of gray mold of tomato fruits was reduced by 77.8% in tomatoes treated with DDHR compared with the control at a concentration of 500 mg L^−1^, and DDHR completely inhibited gray mold development at a concentration of 1000 mg L^−1^. Neither chemicals showed any phytotoxicity toward tomato fruit.

**Table 2:** Disease control efficacy of 32,33-didehydroroflamycoin (DDHR) and fludioxonil against gray mold on tomato fruits caused by *Botrytis cinerea*^a^.

Treatment	Concentration (mg L^−1^)	Disease incidence (%)^b^
Control	0	100.0 ± 0.0^c^ a
DDHR	1	100.0 ± 0.0 a
	10	100.0 ± 0.0 a
	100	85.2 ± 17.0 b
	500	22.2 ± 11.1 c
	1000	0.0 ± 0.0 d
Fludioxonil	1	100.0 ± 0.0 a
	10	77.8 ± 11.1 b
	100	0.0 ± 0.0 d
	500	0.0 ± 0.0 d
	1000	0.0 ± 0.0 d

^a^Conidial suspension (2 × 10^6^ conidia L^−1^) of *B. cinerea* was used as inoculum.

^b^Disease incidence (%) = [(number of infected wounds)/(total wounds per replicate)] × 100.

^c^Mean ± standard deviation indicated by letters are significantly different according to the least significant difference test (n = 3; *P* < 0.05).

### Disease control efficacy of DDHR at various inoculum concentrations

**Figure 3:**
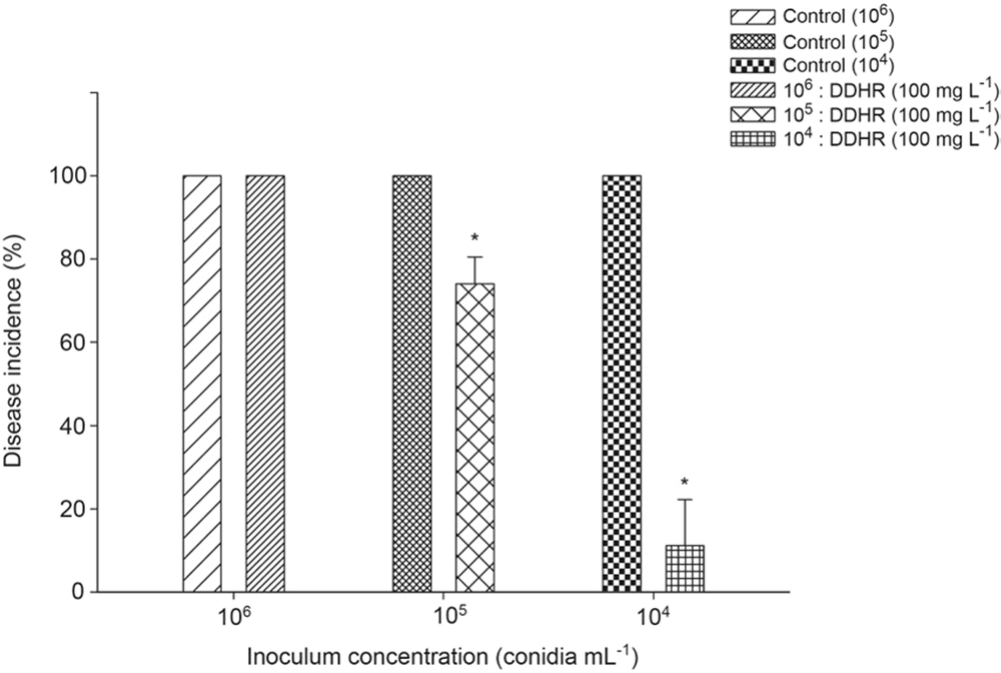
The disease control efficacy of 32,33-didehydroroflamycoin (DDHR; 100 mg L^−1^) at various inoculum concentrations of *B. cinerea*. The disease incidence (%) of the tomato fruits treated with DDHR (100 mg L^−1^) was evaluated 2 d after inoculation with conidial suspensions at three different concentrations (10^4^, 10^5^, and 10^6^ conidia mL^−1^). Bars represent the standard deviation of three replicate experiments. Asterisks (*) indicate significant differences between the control and treatments according to the least significant difference test (n = 3; *P* < 0.05).

The disease control efficacy of DDHR (100 mg L^−1^) at various inoculum concentrations of *B. cinerea* was evaluated (Fig. 3). Two days after inoculation, the control fruits at all inoculum concentrations (10^4^, 10^5^, and 10^6^ conidia mL^−1^) showed a disease incidence of 100%. DDHR (100 mg L^−1^) did not affect gray mold development in the tomato fruits inoculated with 10^6^ conidia mL^−1^, while the disease incidence of the tomato fruits inoculated with 10^5^ conidia mL^−1^ was significantly reduced by 25.9% compared with the control. At the same concentration of DDHR (100 mg L^−1^), a marked reduction in the disease incidence (88.9% compared with the control) was observed in the tomato fruits inoculated with 10^4^ conidia mL^−1^.

### Residue of DDHR in the tomato fruit

**Figure 4:**
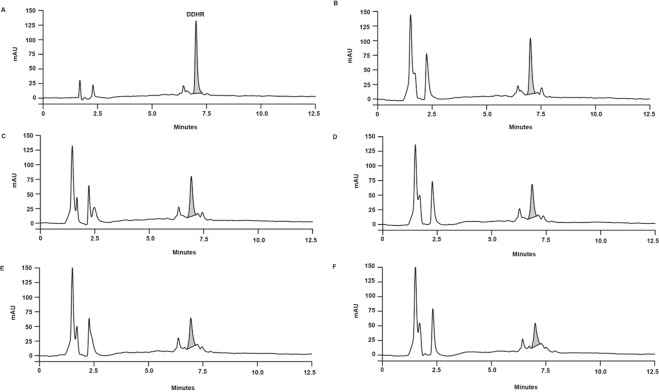
The changes in the quantity of 32,33-didehydroroflamycoin (DDHR) present in tomato fruits over time. (**A**) DDHR (500 mg L^−1^); (**B**) immediately after treatment; (**C**) 6 h after treatment; (**D**) 12 h after treatment; **(E**) 1 d after treatment; and (**F**) 2 d after treatment.

To assess DDHR residue in tomato fruit, a 5 μL aliquot of DDHR (1000 mg L^−1^) was applied to wounded fruits that were extracted with methanol 0, 6, 12, 24, and 48 h after the treatment. The HPLC chromatogram showed that the extraction efficiency of DDHR from treated tomato fruit was approximately 86.8%. The amount of DDHR in the tomato fruit gradually decreased until 2 d. Two days after the treatment, the amount of DDHR in the tomato fruit was significantly reduced by 47.3% compared with that recovered at 0 h after the treatment (Fig. 4). However, fludioxonil remained in the tomato fruit without a significant change in amount for 2 d (Fig. S3).

## Discussion

Polyene macrolides are the most important subgroup of biologically active microbial metabolites^17,18^. Since Hazen and Brown discovered the first polyene antibiotic nystatin in the 1950s, a number of polyenes, including amphotericin B, filipin, natamycin, and rapamycin, have been isolated from *Streptomyces* spp.^19–21^. Nystatin, amphotericin B, and natamycin have been used to treat life-threatening fungal infections or to prevent fungal contamination in clinical practice and the food industry for almost 60 years. It has been reported that the excellent antifungal activity against yeast and various filamentous fungal species of most polyenes discovered so far is due to the disruption of the fungal cell membrane^22–27^. Despite their potent and broad-spectrum antifungal activity, polyenes are not well recognized as fungicides for the control of plant diseases because of their inherent structural vulnerability. Specifically, the polyenes undergo inactivation or degradation under harsh conditions, such as elevated temperature, atmospheric oxygen, and exposure to UV light, which are routinely encountered in crop fields. However, their potent antifungal activities and low residual side effects have encouraged researchers to continue studies on the agricultural use of polyenes as postharvest fungicides^14,25,28,29^. Revisiting the uses of polyenes recently revealed their potential as agents for controlling postharvest diseases. For example, natamycin, used as a food preservative in cheese and sausage, was recently reported to have inhibitory effects on Sigatoka disease, which is a devastating banana disease caused by *F. oxysporum* f. sp. *cubense*^30^. It was also reported that bilayer coatings of chitosan and polyethylene wax microemulsion containing natamycin improved the storability of Hami melon at ambient temperature^31^. More recently, novel polyene–lipopeptide combinations, such as nystatin–lipopeptide and natamycin–lipopeptide, were reported to inhibit the growth of postharvest pathogens, such as *Penicillium* spp. and *Geotrichum* spp. *in vitro*^32^. In addition, other combinations of natamycin with triazole or phosphite were reported to prevent the development of postharvest diseases occurring on various types of fruit, such as banana, mandarin, and strawberry^33,34^. Moreover, the U.S. EPA recently established an exemption from the requirement for tolerance to residues of postharvest fungicide natamycin in or on avocado, kiwi, mango, and pineapples. The safety of natamycin as a postharvest fungicide has been indicated. Reports on the application of antifungal polyenes have renewed interest in their potential as agrichemicals for postharvest diseases.

DDHR, a new member of the polyene macrolide family, was recently isolated from a culture of *S. durmitorensis* strain MS405, and its structure was identified by Stodůlková *et al*. Antitumor and antifungal activities of DDHR against various cancer cell lines and pathogenic fungi have also been reported^15,35,36^. Recently, it was identified that the mechanism of action of DDHR is related to membrane disruption through pore formation in the fungal cell membrane^37^. However, the *in vivo* efficacy of DDHR for controlling gray mold, which is the most important postharvest disease, has not yet been evaluated.

In the present study, DDHR showed a broad antifungal spectrum against plant pathogens, similar to that of other polyene antibiotics, such as filipin III, strevertenes, and rimocidins^14,38,39^. *In vivo* assays revealed that DDHR exerted activity to control gray mold of tomato fruit, which was much lower than that of the commercial fungicide fludioxonil. The incidence of gray mold was markedly reduced by 88.9% in tomatoes treated with DDHR (100 mg L^−1^) compared with the control tomato fruits inoculated with a concentration of 10^4^ conidia mL^−1^. These results show that DDHR is capable of effectively protecting tomato fruit against gray mold and has potential as an agent for controlling postharvest disease even at the inoculum concentration 10^5^ conidia mL^−1^, which is rare in natural environments. The use of postharvest fungicide should be considered with caution because the period between treatment and consumption is short. At two days after treatment, the DDHR residue in tomato fruit was significantly reduced by 47.3% of the amount recovered at 0 h. Additionally, recent studies on nystatin and DDHR have shown that DDHR has low toxicity to human cells^35^. These results suggest that DDHR would be relatively safe for use as a postharvest fungicide, especially for freshly cut fruit that is stored at low temperatures and that is vulnerable to gray mold.

In conclusion, this study showed that DDHR produced by *Streptomyces rectiviolaceus* strain DY46 could inhibit mycelial growth of various plant pathogenic fungi that can cause postharvest disease and could reduce the disease incidence of gray mold of tomato fruit. Although the disease control efficacy of DDHR against gray mold was lower than that of the commercial fungicide fludioxonil, DDHR (100 mg L^−1^) markedly reduced the disease incidence of gray mold of tomato fruit inoculated with a concentration of 10^4^ conidia mL^−1^. Moreover, the DDHR residue in tomato fruit was significantly reduced 2 d after treatment. This is the first study to evaluate the potential of DDHR, a new member of the polyene macrolide family, as a control agent against the postharvest disease gray mold of tomato fruit. Further research on formulations and application methods to improve the safety and activity of DDHR for developing DDHR as a new postharvest fungicide is necessary.

## Materials and Methods

### Identification of strain DY46

16S rDNA sequence analysis was performed to identify the actinomycete strain DY46. Genomic DNA of strain DY46, which was cultured on TSA plate at 28 °C for 3 d, was extracted using the boiling lysis method^40^. PCR mixtures and thermal cycling conditions were carried out as described previously^41^. A Basic Local Alignment Search Tool (BLAST) search of the GenBank database was used for analysis of the 16S rDNA sequence.

Morphological characteristics, such as spore chain morphology, spore size, and spore surface ornamentation, of strain DY46 when cultured on ISP4 medium for 14 d at 28 °C were observed using scanning electron microscopy (SEM). The morphological features of strain DY46 were recorded in accordance with the methods recommended by Waksman, Shirling, and Gottlieb and Bergey’s manual of systematic bacteriology.

### Evaluation of disease control efficacy of the cell extract of strain DY46

The ability of the cell extract of strain DY46 to suppress gray mold of tomato fruit was evaluated. To prepare cell extracts for evaluation of the disease control efficacy, a single colony of strain DY46 was inoculated into 1-L brown Erlenmeyer flasks containing 100 mL of 3% tryptic soy broth (TSB; Difco Laboratories, Detroit, MI). The inoculated flasks were incubated at 28 °C for 3 d on a rotary shaker (200 rpm). After three days, a 1 mL aliquot of the seed culture was transferred into 100 mL of TSB in a 1-L brown Erlenmeyer flask. Each flask was incubated at 28 °C for 5 d on a rotary shaker (200 rpm). The cultured broth (1 L) was centrifuged at 10,000 *g* for 30 min at 4 °C to collect DY46 cells, and the collected cells were extracted using 1 L of methanol. The crude cell extracts were concentrated using a rotary evaporator (Büchi, Flawil, Switzerland) under reduced pressure. The resulting residue was dissolved in water containing 1% methanol and serially diluted to 10, 50, and 100 g L^−1^. One gram of crude extract contained 30 mg of the active ingredient. A conidial suspension of *B. cinerea* D071295 was prepared as the inoculum of gray mold of tomato. The conidia of *B. cinerea* D071295 cultured on potato dextrose agar (PDA; Bacto, Beckton Dickinson and Co., Sparks, MD) at 25 °C for 7 d were harvested using 10 mM KH_2_PO_4_-glucose solution containing 0.05% Tween 80, and the conidial suspension was then filtered through two-layer sterile gauze to remove large mycelial fragments. A conidial suspension (2 × 10^6^ conidia mL^−1^) was used as the inoculum of gray mold. Mature, ripe, and fresh tomato fruit (*Solanum lycopersicum* L. cv. Cherelino) were purchased from a local supermarket. Among the tomato fruits with a consistent size of 3.5 cm in diameter and no visible injury or infection, tomato fruit in stage 6 (red color) of the ripening stages specified by the United States Department of Agriculture (USDA) was selected as the experimental material^42^. The selected tomato fruit were immersed in 2% sodium hypochlorite solution for 1 min and then rinsed in sterilized distilled water. Surface-sterilized tomato fruit air-dried on a clean bench were wounded with a sterile nail (one wound per tomato fruit; 3 mm deep and 3 mm wide)^4,43^. Each wound was treated with 5 μL of the cell extract of DY46 or water containing 1% methanol as the control and then inoculated with 5 μL of conidial suspension of *B. cinerea* D071295 after 1 h. All treatments were performed in three replicates (15 fruit per replicate). The treated tomato fruit were placed in a tightly sealed container with a vial containing 5 mL of sterile distilled water to maintain a high relative humidity (∼95%), then the tightly sealed container was stored at 25 °C for 2 d. The disease incidence (%) in tomato fruit was estimated at 2 d after inoculation.

### Purification of the antifungal compound DY46A

The cell extracts of strain DY46 were prepared from cells cultured in TSB (5 L) as described above. The residue was dissolved in 1 L of water containing 1% methanol. To purify the active ingredients from the crude cell extract, flash column chromatography, including Diaion HP-20 (Mitsubishi Chemical Corp., Tokyo, Japan), and C18 (20–63 µm Lichropep RP-18; Merck, Darmstadt, Germany) column chromatography was performed. The crude cell extract adsorbed on the Diaion HP-20 resin was eluted with stepwise gradients of water and methanol (v/v; 100:0, 80:20, 60:40, 40:60, 20:80, and 0:100) and 1 L of acetone. The paper disk (8 mm in diameter) diffusion assay was used to evaluate the antifungal activities of each fraction against *B. cinerea*. The combined active fractions were dissolved in 500 mL of water containing 1% methanol after concentration *in vacuo* and further purified using C18 flash column chromatography. The compound was eluted with stepwise gradients of water and methanol (v/v; 100:0, 80:20, 60:40, 40:60, 20:80, and 0:100). The active fractions obtained after C18 flash column chromatography were dried and redissolved in 20 mL of methanol. A Varian HPLC system (Agilent Technologies, Santa Clara, CA) equipped with a J′sphere ODS-H80 column (250 × 10 mm i.d., 4 µm) (YMC, Kyoto, Japan) was used for further purification. Chromatography was performed using a linear gradient elution system (0–100% methanol in H_2_O) for 60 min, followed by isocratic elution (100% methanol) for 10 min at a flow rate of 1.5 mL min^−1^. HPLC chromatograms of each peak were monitored at an absorbance of 254 nm and collected under dark conditions. In addition, its antifungal activity against *B. cinerea* was evaluated. The purified antifungal compound was denoted as DY46A.

### Structural elucidation of DY46A

High-resolution mass spectrometry (HRMS) was performed to determine the element composition and molecular weight of purified DY46A using electrospray ionization quadrupole time-of-flight (ESI-Q-TOF) tandem mass spectrometry (Waters, Milford, MA). Additionally, the UV-visible absorption spectrum of DY46A dissolved in methanol was monitored in the wavelength range of 200–600 nm. The NMR spectra of DY46A were recorded using a Varian 500 MHz nuclear magnetic resonance (NMR) spectrometer (Agilent Technologies) at room temperature. The ^1^H NMR (500 MHz) spectrum of DY46A was measured in methanol-*d*_4_ (Cambridge Isotope Laboratories, Andover, MA). The chemical shifts were represented by *δ* values (ppm) referenced to the residual proton of methanol-*d*_4_ (3.31 ppm). Coupling constants (*J*) were given in Hz. The ^13^C NMR spectrum (125 MHz) of DY46A was recorded in methanol-*d*_4_. Furthermore, 2D-NMR spectroscopic analysis, including heteronuclear multiple-bond connectivity (HMBC), was performed. Based on the spectroscopic data obtained by these methods, the structure of DY46A was determined.

### Bioassays for the antifungal activity of DY46A

The antifungal activity of all fractions obtained during the entire purification procedure was evaluated against *B. cinerea* using the paper disk agar diffusion method. The conidia of *B. cinerea* were harvested in sterilized water, and the conidial suspension was filtered through two-layer sterile gauze to remove large mycelial fragments. One milliliter of conidial suspension (10^7^ conidia ml^−1^) was added with 100 ml of molten PDA (0.7% agar) at 40 °C and was then poured into sterile 90-mm Petri dishes. The paper disks treated with each fraction were placed on the agar surface.

The modified Clinical and Laboratory Standards Institute (CLSI) broth microdilution method (M38-A Document) was used to determine the minimum inhibitory concentrations (MICs) of DY46A and nystatin (positive control) (MBcell, LA, CA) against various plant pathogenic fungi^14,16^. Twenty-five microliters of quadruple-strength potato dextrose broth (PDB) per well was added to 96-well plates (No. 30096; SPL Life Sciences Inc., Pocheon, Korea), followed by the addition of 25 μl of conidial or zoospore suspension obtained from various plant pathogenic fungi (*Alternaria brassicicola*, *A. mali*, *Botrytis cinerea*, *Cladosporium cucumerinum*, *Colletotrichum coccodes*, *C. gloeosporioides*, *C. orbiculare*, *Cylindrocarpon destructans*, *Fusarium oxysporum* f. sp. *cucumerinum*, *F. oxysporum* f. sp. *lycopersici*, *Penicillium expansum*, *P. capsica*, and *Rhizopus stolonifer* var. *stolonifer*) into each well to a final concentration of 4 × 10^4^ spores mL^−1^. Finally, DY46A and nystatin were added to each well at a final concentration of 1–128 mg L^−1^. MICs against a variety of plant pathogenic fungi were determined by visual inspection of complete growth inhibition in accordance with the CLSI M38-A method.

### Evaluation of disease control efficacy of DY46A

The protective activities of DY46A and commercial fungicide fludioxonil against gray mold of tomato fruit were evaluated. The commercial fungicide fludioxonil and DY46A were dissolved in water containing 1% methanol and serially diluted to 1, 10, 100, 500, and 1000 mg L^−1^. The conidia of *B. cinerea* D071295 cultured on PDA at 25 °C for 7 d were harvested with 10 mM KH_2_PO_4_-glucose solution containing 0.05% Tween 80. A conidial suspension (2 × 10^6^ conidia mL^−1^) of *B. cinerea* D071295 was prepared as the inoculums of gray mold. Mature, ripe, and fresh tomato fruits (*Solanum lycopersicum* L. cv. Campari) were purchased from a local supermarket. Among tomato fruits (5 cm in diameter) with no visible injury or infection, the fruits in stage 6 (red color) of the ripening stages as specified by the United States Department of Agriculture (USDA) were selected for the experiment^42^. They were then immersed in 2% sodium hypochlorite solution for 1 min and then rinsed in sterilized distilled water. Three wounds (3 mm deep and 3 mm wide) per fruit were made using a sterile nail at the equator after the surface-sterilized tomatoes were air-dried on a clean bench^4^. Each wound was treated with 5 μL of the chemical solutions or water containing 1% methanol as the control and was then inoculated with 5 μL of conidial suspension of *B. cinerea* D071295 after 1 h. All treatments were performed in three replicates (three fruit per replicate). The treated tomato fruit were placed in a tightly sealed container with a vial containing 5 ml of sterile distilled water to maintain a high relative humidity (∼95%), then the tightly sealed container was stored at 25 °C for 2 d. The disease incidence (%) in tomato fruit was estimated at 2 d after inoculation.

### Disease control efficacy of DDHR at various inoculum concentrations

The disease control efficacy of DDHR (100 mg L^−1^) at various inoculum concentrations of *B. cinerea* was investigated as follows: Surface-sterilized tomato fruit air-dried on a clean bench were wounded with a sterile nail (one wound per tomato fruit; 3 mm deep and 3 mm wide) as described above^4,43^. The conidial suspensions (10^4^, 10^5^, and 10^6^ conidia mL^−1^) of *B. cinerea* D071295 were prepared as the inoculums of gray mold. Each wound was treated with 5 μL of DDHR (100 mg L^−1^) or water containing 1% methanol as the control and then inoculated with 5 μL of conidial suspensions (10^4^, 10^5^, and 10^6^ conidia mL^−1^) of *B. cinerea* D071295 after 1 h. All treatments were performed in three replicates (three fruit per replicate). The treated tomato fruits were placed in a sealed container with a vial containing 5 ml of sterile distilled water to maintain a high relative humidity (∼95%), then the tightly sealed container was stored at 25 °C for 2 d. The disease incidence (%) in tomato fruits was estimated at 2 d after inoculation.

### Residue of DDHR in tomato fruit

The residue of DDHR in tomato fruit was investigated as follows: Surface-sterilized tomato fruit were wounded using a sterile nail (one wound per tomato fruit; 3 mm deep and 3 mm wide). Each wound was then treated with 5 μL of DDHR (1000 mg L^−1^). All treatments were performed in three replicates. At 0, 6, 12, 24, and 48 h after the treatment, the tomato fruit was extracted with methanol. Then, two-fold-diluted methanol extracts were analyzed using the Varian HPLC system (Agilent Technologies) equipped with a J′sphere ODS-H80 column (150 × 4.6 mm i.d., 4 µm) (YMC). Chromatography was performed using a linear gradient elution system (20%–100% acetonitrile in H_2_O) for 10 min, followed by isocratic elution (100% acetonitrile) for 5 min at a flow rate of 1 mL min^−1^. DDHR was monitored at an absorbance of 330 nm. The residue of the commercial fungicide fludioxonil in the tomato fruit was investigated as described above. Fludioxonil was monitored at an absorbance of 254 nm. Each extract was analyzed in three replicates, calculating the average sample area.

### Statistical analysis

Statistical analyses were carried out using Statistical Analysis Systems version 9.2 (SAS Institute, Cary, NC) at a 5% significance level. All data are expressed as the mean ± standard deviation, and the least significant difference (LSD) test for multiple comparisons was conducted to determine the significant differences between the mean values.

## Supplementary information

Supplementary Data

## Data Availability

All data generated or analyzed during this study are included in this published article and its Supplementary Information Files.
