# Supplementary material for: Disease control efficacy of 32,33-didehydroroflamycoin produced by Streptomyces rectiviolaceus strain DY46 against gray mold of tomato fruit
Source: Sci Rep. 2019 Sep 19;9:13533. doi: 10.1038/s41598-019-49779-6 (PMC6753085; doi:10.1038/s41598-019-49779-6)
Supplement: Supplementary file 1 — Supplementary Data [file 41598_2019_49779_MOESM1_ESM.docx]

**Disease control efficacy of 32,33-didehydroroflamycoin produced by *Streptomyces rectiviolaceus* strain DY46 against gray mold of tomato fruit**

**Jeong Do Kim^a^, Min Young Park^b^, Byeong Jun Jeon^b^, Beom Seok Kim^b,c,^***

**^a^** Korea Institute of Science and Technology (KIST) Gangneung Institute, Gangneung, 25451, Republic of Korea

**^b^** Department of Biosystems and Biotechnology, Korea University Graduate School, Seoul, 02841, Republic of Korea

**^c^** Division of Biotechnology, College of Life Sciences and Biotechnology, Korea University, Seoul, 02841, Republic of Korea

***Corresponding author:** Prof. Dr. Beom Seok Kim

Division of Biotechnology, College of Life Sciences and Biotechnology, Korea University, Seoul, 02841, Republic of Korea

**E-mail:** [biskim@korea.ac.kr](mailto:biskim@korea.ac.kr)

**Phone:** 82-2-3290-3047

**Fax:** 82-2-925-1715

**Supplementary data**

**Table S1**

^13^C nuclear magnetic resonance spectra of DY46A (125 MHz for ^13^C; CD_3_OD; 25 °C)

| *Atom no.* | *^δ^C* | |
| --- | --- | --- |
|  | 32,33-didehydroroflamycoin | DY46A |
| 1 | 170.1 | 169.9 |
| 2 | 127.9 | 127.7 |
| 3 | 140.8 | 140.1 |
| 4 | 129.0 | 128.8 |
| 5 | 141.9 | 141.8 |
| 6 | 133.8 | 133.7 |
| 7 | 138.7 | 138.5 |
| 8 | 132.3 | 132.2 |
| 9 | 137.4 | 137.3 |
| 10 | 133.8 | 133.6 |
| 11 | 135.6 | 135.5 |
| 12 | 40.9 | 41.3 |
| 13 | 69.4 | 69.3 |
| 14 | 42.3 | 42.2 |
| 15 | 65.4 | 65.3 |
| 16 | 46.0 | 46.1 |
| 17 | 99.1 | 99.0 |
| 18 | 49.8 | 49.9 |
| 19 | 66.3 | 66.2 |
| 20 | 46.2 | 46.2 |
| 21 | 66.8 | 66.5 |
| 22 | 46.3 | 46.4 |
| 23 | 68.8 | 68.6 |
| 24 | 46.8 | 46.7 |
| 25 | 68.1 | 68.3 |
| 26 | 46.0 | 46.1 |
| 27 | 69.3 | 69.1 |
| 28 | 45.8 | 45.6 |
| 29 | 71.7 | 72.3 |
| 30 | 46.4 | 46.6 |
| 31 | 71.4 | 71.5 |
| 32 | 133.5 | 133.3 |
| 33 | 133.5 | 133.3 |
| 34 | 37.9 | 37.7 |
| 35 | 82.4 | 82.2 |
| 36 | 31.1 | 31.0 |
| 2-Me | 13.3 | 13.1 |
| 34-Me | 12.2 | 12.0 |
| 36-Me-u | 20.6 | 20.4 |
| 36-Me-d | 19.6 | 19.4 |


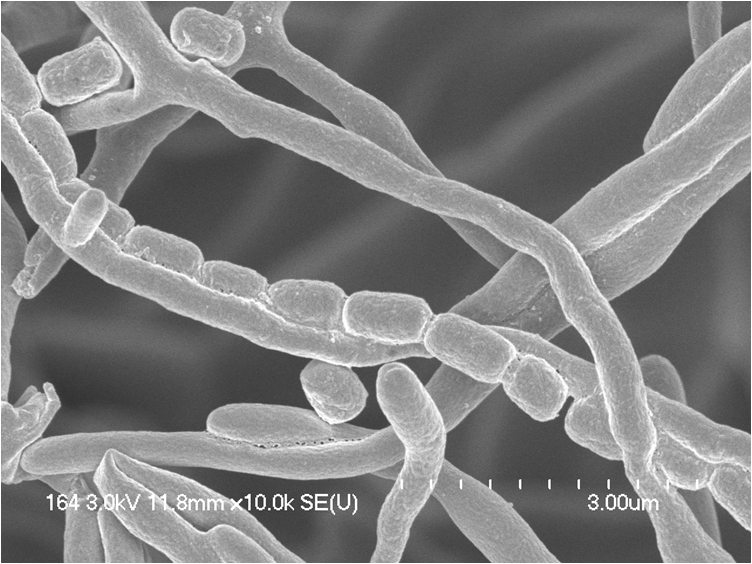


**Fig. S1.** Scanning electron micrograph of *Streptomyces rectiviolaceus* strain DY46 cultured on ISP4 for 14 d at 28 °C

**
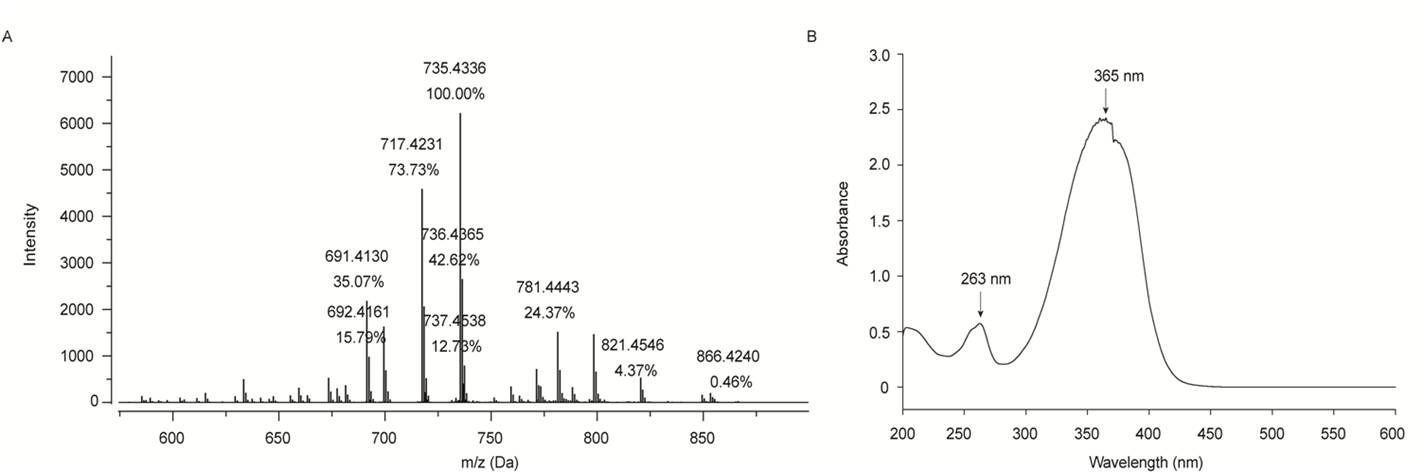
**

**Fig. S2.** Mass spectra (A) and UV spectroscopic data (B) of the polyene antibiotic DY46A

**
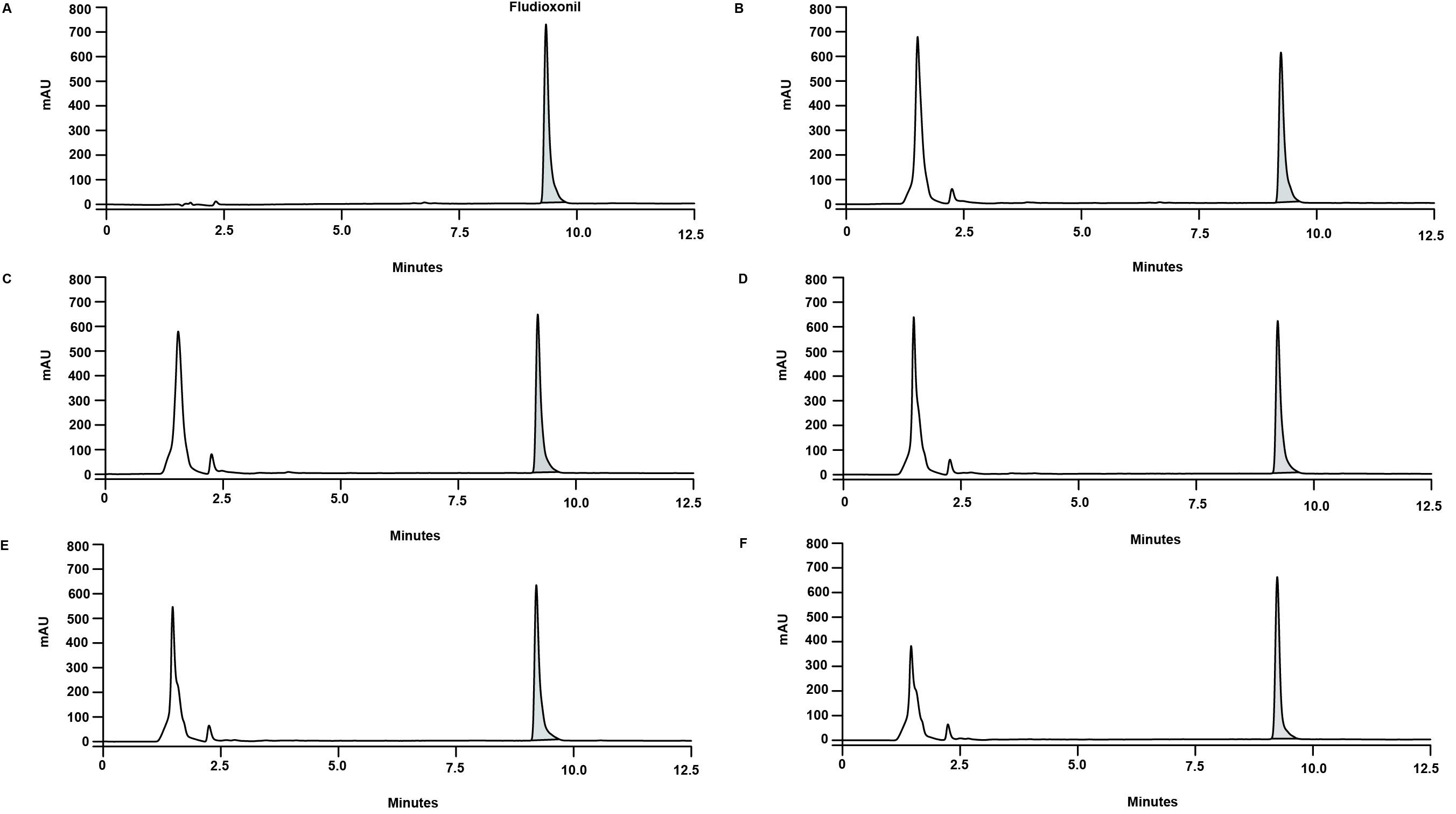
**

**Fig. S3.** The change of fludioxonil residue according to time in the tomato fruit. (A) fludioxonil (500 mg L^−1^); (B) immediately after treatment; (C) 6 h after treatment; (D) 12 h after treatment; (E) 1 d after treatment; and (F) 2 d after treatment
